# Methodological bias in cluster randomised trials

**DOI:** 10.1186/1471-2288-5-10

**Published:** 2005-03-02

**Authors:** Seokyung Hahn, Suezann Puffer, David J Torgerson, Judith Watson

**Affiliations:** 1 Medical Research Collaborating Center, Seoul National University College of Medicine, 2nd Floor Cancer Research Institute Building, 28 Yongon Dong, Chongno Gu, Seoul 110-744, Korea; 2 York Trials Unit, Department of Health Sciences, York YO10 5DD, UK

## Abstract

**Background:**

Cluster randomised trials can be susceptible to a range of methodological problems. These problems are not commonly recognised by many researchers. In this paper we discuss the issues that can lead to bias in cluster trials.

**Methods:**

We used a sample of cluster randomised trials from a recent review and from a systematic review of hip protectors. We compared the mean age of participants between intervention groups in a sample of 'good' cluster trials with a sample of potentially biased trials. We also compared the effect sizes, in a funnel plot, between hip protector trials that used individual randomisation compared with those that used cluster randomisation.

**Results:**

There is a tendency for cluster trials, with evidence methodological biases, to also show an age imbalance between treatment groups. In a funnel plot we show that all cluster trials show a large positive effect of hip protectors whilst individually randomised trials show a range of positive and negative effects, suggesting that cluster trials may be producing a biased estimate of effect.

**Conclusion:**

Methodological biases in the design and execution of cluster randomised trials is frequent. Some of these biases associated with the use of cluster designs can be avoided through careful attention to the design of cluster trials. Firstly, if possible, individual allocation should be used. Secondly, if cluster allocation is required, then ideally participants should be identified before random allocation of the clusters. Third, if prior identification is not possible, then an independent recruiter should be used to recruit participants.

## Background

The randomised controlled trial (RCT) has a number of important features that make it the 'gold-standard' evaluation method. One of the most important aspects of random allocation is that it eliminates selection bias. Randomisation ensures that the two or more groups formed are similar, except for chance differences, in all aspects. Nevertheless unless trials are undertaken in a rigorous manner biases can be introduced that negate the effect of random allocation. Indeed, a poorly conducted RCT can be worse than a good observational study as the latter is interpreted in the light of possible confounding whereas the results of an RCT might be uncritically accepted.

Random allocation can take place either at the level of the individual level or at a higher group or cluster level. In a cluster randomised trial groups of people are allocated to receive an intervention or not. In some areas of evaluation (e.g., education) the cluster is the natural method of allocation. For example, a trial among school children may well randomise by class or by school rather than by individual child. Allocation by cluster may be preferable for a number of reasons. There may be practical reasons: for instance, teaching a novel curriculum will be easier to use existing classes than form new ones through randomisation. There may be contamination issues. Individuals allocated to a control treatment may inadvertently receive some aspects of the intervention if they are in proximity to the treated group.

Allocation by cluster has some important statistical issues that have been addressed 65 years ago in the educational trial literature [1] and subsequently widely in medical statistics [2]. In brief, analysis of cluster trials needs to take into account the clustered nature of the data otherwise the risk of a Type I error (i.e., erroneously concluding there was a statistically significant difference) increases. However, more seriously in our view is the potential of cluster trials producing a biased estimate of treatment effect.

Randomisation should eliminate selection bias. Selection bias can be reintroduced within any trial if there is high loss to follow-up or failure to use intention to treat analysis. In cluster trials selection bias can also be introduced through participant recruitment. Because cluster trials often recruit their participants after the clusters have been randomly allocated this can lead to selection effects[3,4]. There are a number of potential reasons for this.

### Foreknowledge of allocation

If the person recruiting participants has both knowledge of the clinical characteristics of the participants and of the allocation schedule biased recruitment can occur. Subversion, within individually randomised trials, can occur by recruiting participants with poor prognostic characteristics so that they are more likely to enter the 'unfavoured' group [5,6]. Evidence for the biasing effects of allocation foreknowledge has been shown on treatment effect sizes [7,8]. Consequently a rigorously designed individually randomised trial ought to conceal the allocation schedule from the people who are recruiting participants.

Cluster randomised trials often do not, or cannot, conceal treatment allocation. For example, a trial was undertaken to reduce violence among children randomised by school [9]. After allocation the children were recruited into the study and the intervention was delivered. The allocation could not be concealed from the teachers researchers or children. This has two potentially unfortunate consequences. Awareness of the allocation can lead to biased recruitment in cluster trials [10]. Alternatively, or in addition, participants can differentially refuse consent to participate in the trial and this could be another source of selection bias. For example, in a cluster trial evaluating the use of advanced end of life directives among residents of nursing homes there was a differential in participant rates of 83% among people in the intervention homes compared with 92% in the control arm [11]. Such differential participation rate can lead to selection bias.

### Treatment effects on recruitment

Recruitment of participants with different clinical characteristics is not necessarily a sign of subversion it could be simply a consequence of the cluster level intervention. For example, in an evaluation of an educational package for the treatment of back pain primary care physicians were trained in 'evidence based' management of back pain [12]. This training was associated with an increased recruitment rate among practices allocated to training compared with no training. Because training involved recognition and diagnosis of back pain with hindsight we should not be unsurprised that differential recruitment would occur in this instance.

As well as having more potential, than individually randomised trials, for the introduction of selection bias. Cluster randomised trials also may be at more risk of dilution bias. Because consent for treatment is often not obtained until after randomisation more participants, than in an individually randomised study, may refuse treatment and this will consequently dilute any treatment effects. For example, Kendrick and colleagues in a cluster randomised trial to prevent accidental injuries among young children found that only 75% of the group allocated to the experimental group actually received the intervention [13].

**Figure 1:**
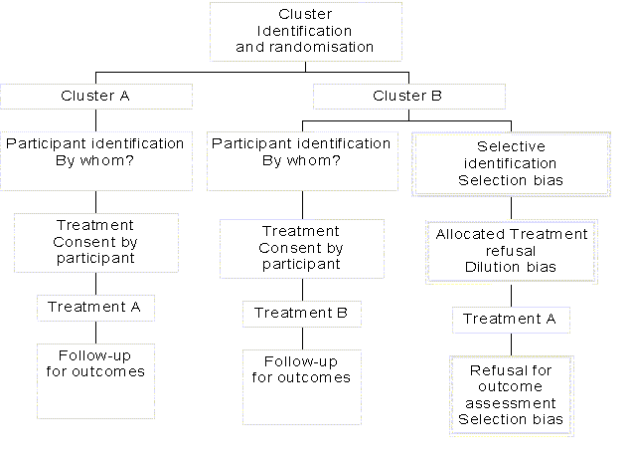
Sources of bias in cluster trials

Whatever the underlying reasons for differences in recruitment the consequences are potentially the same: selection bias has been introduced. Figure 1 shows the potential sources of bias that can occur after cluster randomisation. With the introduction of selection bias trial results are unreliable. In this paper we examine some evidence for this phenomenon and make recommendations on how to design this problem out of future cluster trials.

#### Evidence for recruitment bias among individual trials

A recent review identified a sample of 36 cluster randomised trials from three major general medical journals, between 1997 and 2002 [4]. This review identified all cluster randomised trials published in three major medical journals over a period of five years. In this review 15 of the trials could have experienced bias in their recruitment of participants. Of these 15 trials seven showed some evidence in the published papers of consenting differential numbers of participants or excluding participants in a selective fashion. One of the remaining 8 trials, whilst having no evidence of bias in the original published paper was later subsequently found to have experienced recruitment bias [10]. Therefore, 25% of cluster trials published in major clinical journals suffered potential selection bias.

On the other hand a review of 152 cluster trials undertaken in primary care found that only 8 (5%) were found were the authors reported differential recruitment [14]. However, unlike Puffer and colleagues each trial was not carefully scrutinised to ascertain whether or not there was a problem of biased recruitment (Eldridge, personal communication).

**Figure 2:**
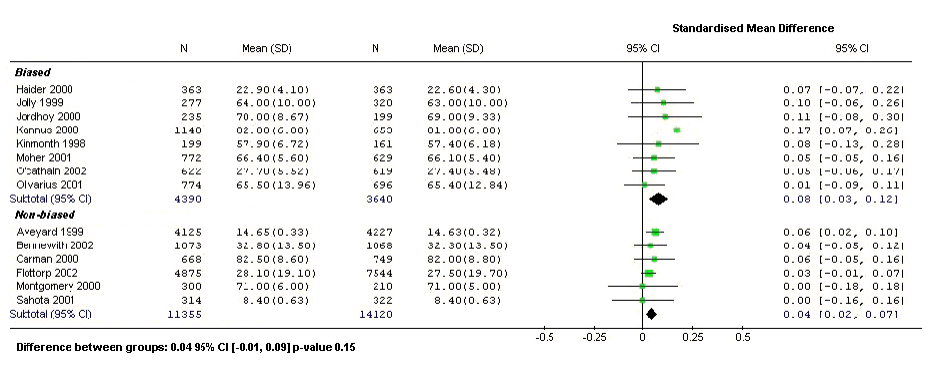
Standardised mean differences of patient age

Although Puffer and colleagues noted that some trials had significant differences in recruitment and retention rates between groups they did not investigate whether or not this had an impact on important treatment covariates. To assess whether observed differences in recruitment could have had an effect on important predictors of outcome we examined the age differences between treatment groups. We chose age for two reasons: first, it is a commonly reported baseline characteristic and, second, is the most likely common confounder across different disease groups. Nevertheless, we acknowledge that biased recruitment may not manifest itself in terms of age differences [12]. From the 36 trials we identified 14 that reported, either directly or indirectly, the mean age and standard deviation of the treatment groups. Of the 14 trials that were included nine stated they had taken clustering into account in their sample size calculation, two had not and the remaining three it was not clear whether they had adjusted their sample size. We then grouped the 14 trials according to whether Puffer *et al *considered there was evidence for differential recruitment. Eight out of the 14 trials had been regarded as potentially biased. In Figure 2 we plot the standardised mean age differences between treatment groups (ie., age difference divided by the pooled within group standard deviation). Negative age differences were all converted to positive differences as we were uninterested in the direction of the bias.

As can be seen in Figure 2 the age difference between treatment groups tend to be larger in the *potentially *biased group. The mean age difference was greater than 10% of their standard deviation in 3 out of the 8 potentially biased trials. The pooled standardised mean difference in the biased group was also twice as large as that in the non-biased group. A test for the difference using a meta-regression resulted in a non-significant p-value of 0.15; therefore, the difference observed in this instance was not conclusive. Age imbalances for any single trial could be due to chance as it is more difficult to achieve balance in cluster randomised trials compared with individually randomised trials due to the smaller number of allocated units. On the other hand, cluster trials, like individually randomised trials should be balanced across *all *cluster trials if there was no bias present.

**Figure 3:**
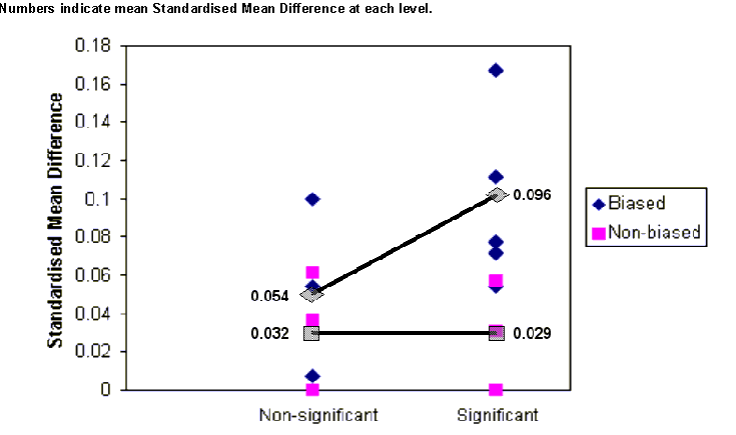
Standardised mean difference by bias group and treatment significance

In Figure 3 we plot the standardised mean age differences by whether or not the trial showed a statistically significant effect. The significance was determined as p-value < 0.05. In all 14 trials the analysis took the clustering effect into account through various methods The figure suggests that significant trial results were associated more often with potentially biased recruitment with larger baseline differences in age, even though a formal test for interaction did not show a statistical significance (p-value 0.3) as this was not adequately powered.

#### Evidence for bias from a systematic review

Cluster randomised trials often answer different questions to individually randomised trials or cannot use individual allocation. Therefore, it is difficult to make a direct comparison between individual and cluster randomised trials in terms of the likely differences in effect sizes within the same subject area. However, within the area of hip protection for fracture prevention there are trials using both individual and cluster allocation. The most recent Cochrane review of hip protectors has identified 13 RCTs of hip protectors with hip fracture outcomes [15]. In addition, there is a large individually randomised trial that has not yet been included in the review (i.e., 14 in total) [16].

**Figure 4:**
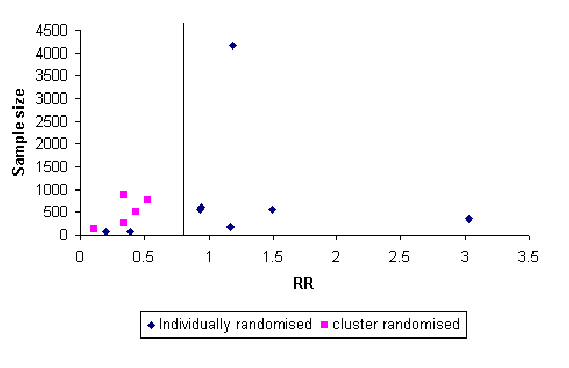
Funnel plot of individually and cluster randomised trials

In figure 4 the effect sizes from these trials are plotted against their sample size. The sample sizes were adjusted for the design effect for the cluster trials and therefore the sample sizes for these trials are the effective sample sizes (sample size divided by the design effect). One out of the five cluster randomised trials reported their design effect, from which we estimated an intra-cluster correlation coefficient (ICC) and applied this to the other four studies, as they are all similar trials, to calculate a correction factor. As the figure shows the resulting funnel plot indicates little evidence of effect from individually randomised trials. In contrast, all of the cluster trials show a substantial benefit when using the cluster design. This suggestion of bias could be as a result of publication bias. On the other hand, there are a number of alternative explanations. First, the cluster trials might have been undertaken in a different setting than the individually randomised trials and this might account for the observed differences in effect. Second, the intervention (hip protection) may work better using a clustered design. Third, there might be treatment contamination in the individually randomised trials: biasing the treatment effect towards the null. Fourth, the observed differences may be due to poor implementation of cluster trial methodology, which biases the results of those trials towards the positive.

There is a tendency for the cluster trials to be largely undertaken among residents of nursing homes compared with individually randomised trials – although one of the largest individually randomised trial was in a nursing home setting. It is possible that compliance might have been better in a nursing home setting and this could account for the difference in effect. However, the compliance rates were not that different from those trials using individually randomisation.

The second reason that hip protection might work in a clustered design might be that the intervention is delivered as a 'package' of care and their use alerts the clinical staff responsible (e.g., nursing home staff) to the dangers of falls and this encourages other anti-fracture interventions.

It is possible that in the individually randomised trials the control group could have been 'contaminated' by accessing hip protection by, for example, buying the product themselves. In a large individually randomised trial we undertook of hip protectors [16] some participants in the control arm did purchase hip protectors; however, the prevalence of this was very low.

An alternative explanation of the difference was poor implementation of the cluster trial methodology: including selective recruitment, differential loss to follow-up and failure to use intention to treat analysis. For example, the largest cluster randomised trial had a 30% difference in the population that were included in the trial after random allocation [17].

#### Preventing biased recruitment

In order for cluster randomised trials to provide unbiased evidence for treatments we must design out any sources of recruitment bias. In this section we will consider design suggestions that should minimise this threat.

#### Use individual allocation

Often cluster randomisation is used to overcome the *perceived *threat of contamination between the treatment groups. Although in many instances this threat is real in some cases there may be little contamination. Indeed, even if there are quite high contamination rates (e.g., 20%) it may still be more efficient in sample size terms to randomise more patients in an individual trial and accept a diluted effect size [3]. Therefore, one solution to avoiding biased recruitment is to avoid using cluster trial methods if at all possible.

#### Prior identification of participants

In some instances it may be possible to identify participants before cluster allocation. For example, if we consider a school based evaluation of a health promotion curriculum. Children within schools or intact classes can be identified before the cluster allocation. Children and their parents can be asked to participate in the study and are presented with the alternatives under consideration. Once consent has been obtained to take part in the study then the schools or classes are randomised to the different curricula.

#### Independent recruitment

Evaluation of an intervention for incidence disease cases means prior identification is not possible. For example, consider a trial of educating primary care physicians for the treatment of acute shoulder pain. Because the condition has an incident nature it is necessary to recruit participants in a prospective manner. Should the primary care physician undertake this then selection bias is likely to ensue. Therefore, to reduce this possibility an 'independent' person needs to recruit participants. Consider a recent example of such an approach. In a trial of educating GPs for the identification and treatment of depression in primary care trial participants were recruited by practice receptionists. Because the receptionists from both intervention and control practices had been exposed to the same amount of trial training then the potential for selection bias is reduced, although never eliminated [18].

## Discussion

The use of cluster randomised trials has significantly increased in medical research in recent years [19]. Despite Lindquist outlining an appropriate approach to the analysis of cluster trials in 1940 [1] – many fail to undertake the analysis taking the clustering effect into account. Consequently the attention of many medical statisticians has been directed at the appropriate analysis and sample size issues with less attention to more serious problems with the design of cluster trials. Whilst inappropriate analysis will give misleading precision (i.e., smaller confidence intervals and lower p values) it will rarely change the point estimate of a treatment effect. In contrast, bias can give a misleading effect size estimate.

Cluster randomised trials are potentially more susceptible to some forms of bias than individually randomised trials. Biased recruitment can be a problem in some cluster randomised trials. One symptom of biased recruitment is differential recruitment rates. However, one trial noted significant selection bias even when there were similar recruitment rates [10]. Therefore, even when recruitment rates appear similar between treatment arms selection bias can be introduced. We have examined the issue of bias in cluster trials by comparing a sample of trials against similar individually randomised studies from a review of hip protectors. This suggested a difference in effect size that was dependent upon the type of study design. However, the sample size was small and there are alternative explanations to the apparent differences in effect sizes: not least the explanation of chance. We have also looked at baseline differences in ages of people in cluster trials that appeared to be free of bias with those that seem to have had bias introduced due to poor methodological application of design. There was a difference in age imbalance, which was suggestive of an interaction with statistical significance of trial results although a formal statistical test failed to show a significance of the difference. Our sample size was relatively small and we could have missed a statistically significant difference through lack of statistical power. Nevertheless, this paper does raise concerns about the design of cluster trials and signals that such trials should be used with caution.

If there are important confounding variables, stratification, matching or regression models for clustered data are required. Studies with evidence of biased recruitment might try methods of analysis that allow for observed confounding. For example, if there were imbalances in patient or cluster level covariates between the randomised groups multi-level or hierarchical models explicitly model the treatment effect adjusting for the confounding, provided that there is a fairly large number of clusters. However, even the most sophisticated statistical analysis cannot adjust for the unmeasured or unknown confounder, which is one of the main reasons we undertake random allocation. Therefore, it is crucial that we avoid the introduction of bias into our cluster designs.

Future cluster randomised trials should endeavour to either identify participants before randomisation or use an independent person, preferably blind to allocation, to recruit participants. Furthermore, cluster randomised trials ought to be undertaken such that loss to follow-up is similar between groups and intention to treat is always used.

## Competing interests

The author(s) declare that they have no competing interests.

## Authors' contributions

SH undertook the statistical analysis and wrote sections about statistical methods and implications. SP wrote the first draft and contributed to the original data collection. DT had the original idea of the paper and undertook revisions to the original draft. SW contributed to the original review and collected additional data for the paper. All authors contributed to commenting on drafts of the manuscript.

## Pre-publication history

The pre-publication history for this paper can be accessed here:


